# A Different Perspective on Evaluating the Malignancy Rate of the Non-Diagnostic Category of the Bethesda System for Reporting Thyroid Cytopathology: A Single Institute Experience and Review of the Literature

**DOI:** 10.1371/journal.pone.0162745

**Published:** 2016-09-14

**Authors:** Pembegul Gunes, Sule Canberk, Mine Onenerk, Murat Erkan, Nilufer Gursan, Emine Kilinc, Gamze Zeynep Kilicoglu

**Affiliations:** 1 Department of Pathology, Haydarpasa Numune Education and Research Hospital, Istanbul, Turkey; 2 Department of Interventional Radiology, Haydarpasa Numune Education and Research Hospital, Istanbul, Turkey; University of Toronto, CANADA

## Abstract

**Objective:**

To determine the malignancy rate in the non-diagnostic (ND) category of the Bethesda System for Reporting Thyroid Cytopathology (BSRTC) based on a different approach in relation to histopathology diagnoses.

**Study Design:**

All ND fine needle aspirations (FNAs) that were performed under ultrasound guidance by an interventional radiologist with rapid on-site evaluation were included in the study. Slides were reevaluated to identify the cause of inadequacy as “qualitative” or “quantitative.” The malignancy rate of the ND category was assessed. Nodule/patient characteristics were compared between benign and malignant cases within the study cohort.

**Results:**

The study cohort consisted of 192 ND aspirations. Overall there were 156 (81.3%) women and 36 (18.7%) men with a mean age of 50.6 years (range 24–82 years). The malignancy rate was 4.7%. None of the nodules (size, consistency, and number) or patient characteristics (gender and age) were found to be predictive of malignancy.

**Conclusion:**

The malignancy rate of the ND category was high when compared to BSRTC predictions, but at the low end of the reported malignancy rates in the literature. Our results revealed that cyto-histopathologic correlation and method of malignancy rate estimation could have an effect on a wide range of reported malignancy rates. Furthermore, patient/nodule dependent factors were not statistically found to be predictive of malignancy.

## Introduction

The most accurate and cost-effective combination for differentiating malignant from benign thyroid nodules is measurement of serum thyroid stimulating hormone, thyroid ultrasound, and fine-needle aspiration (FNA) [1]. Considering FNA, obtaining inadequate specimen is a well-known limitation that is referred to as non-diagnostic (ND) according to the Bethesda System for Reporting Thyroid Cytopathology (BSRTC). Excluding samples composed exclusively of cystic content, it should be limited to less than 10% of an institution’s thyroid FNA specimens [2]. However, it was reported as 0.7%-23% in the literature, which is higher than the recommended percentage [1–7].

The American Thyroid Association (ATA) and American Association of Clinical Endocrinologists (AACE) recommend repeat FNA after an ND result. Surgical excision or close observation is considered based on sonographic pattern and accompanying clinical risk factors for repeat ND nodules [1].

Although BSRTC predicted the overall malignancy risk as 1–4%, reported malignancy rates demonstrate significant variability between 2% and 70.6% [2, 4, 6, 8–14]. Most nodules in the ND category are benign, with only a small percentage that are eventually resected; thus, in some studies, accuracy was determined in relation to the results of repeat FNAs [8, 9, 15, 16]. However, considering the false positive/negative rates of the FNA method, evaluation of accuracy should be based on cyto-histopathologic comparison where available. Moreover, the ND category is not homogenous in terms of subcategories including “acellular/hypocellular,” “cyst fluid only,” and “smears with poor preparation technique;” thus, all might have a different effect on the malignancy rate.

The aims of this study are to estimate the malignancy rate of the ND category that was subcategorized as “qualitative” or “quantitative” with the use of BSRTC criteria, and to identify patient/nodule characteristics that are associated with malignancy in nodules with an ND result.

## Materials and Methods

### Study cohort

All thyroid FNAs were retrieved from the medical records of Haydarpasa Numune Education and Research Hospital between January 1999 and July 2014. ND FNAs with subsequent thyroidectomy were included in this study. Adequacy was evaluated according to BSRTC after 2009 [4]. Before 2009, cases diagnosed as “non-diagnostic”, “cyst fluid” and “acellular material”, according the criteria proposed by Goellner et al [17] were accepted as the equivalent of the ND category of the BSRTC

Medical records of all cases were reviewed for demographics (age and gender), ultrasound features (size, number, location, and consistency of the nodules), clinical findings (thyroid hormone levels), and cytological/pathological variables.

### Subcategorizing the ND category of the BSRTC

**Fig 1:**
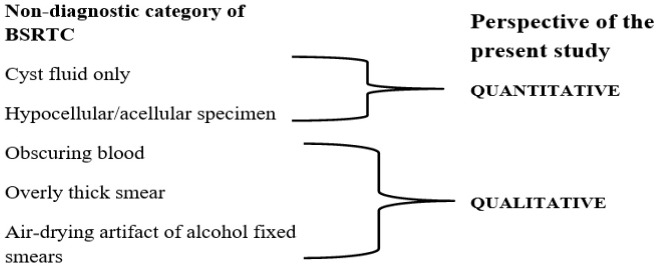
Subcategorizing the ND category of the BSRTC.

All slides from ND cases were re-reviewed by a cytopathologist (S.C) and a pathologist (P.G) to subcategorize as qualitative ND or quantitative ND. Quantitative criteria are based on the number of follicular cells and cell groups: acellular specimen, less than six groups consisting of at least ten benign follicular cells, and the presence of histiocytes (cyst fluid) only. Air-drying artifacts of alcohol-fixed smears that obscure the blood and thick slides that prevent interpretation constitute the qualitative group of the ND category [4] (Fig 1).

### Fine-needle aspiration procedure

All FNAs were performed using 22-gauge needles, and in each case a maximum of three passes were made. Two direct smears were prepared for each aspiration. Air-dried Diff-Quik stained smears were used for rapid on-site evaluation, and one slide was fixed in 95% alcohol for Papanicolaou staining. Cells blocks were made from the excessive material in the syringe.

### Inclusion and exclusion criteria

All the cases in the ND category that had undergone FNA under ultrasound guidance by an interventional radiologist with accompanying rapid on-site evaluation (ROSE) were included in the study. FNAs that did not meet these criteria were excluded from the study cohort. ND cases were included only if the ND aspiration was the “initial” (first aspiration of a nodule) result of a specific nodule.

### Patient management for ND cytology

Our institutional protocol recommends a repeat FNA after a 3-month waiting period for an initial ND result. If there was a strong suspicion of malignancy based on clinical findings, the presence of significant cosmetic deformity/ pressure on cervical structures, or simply patient refusal of a repeat FNA, surgeons might consider surgery [3]. A large nodular goiter, which is endemic in Turkey, also constitutes a common indication of surgery. Due to the Chernobyl Nuclear Power Plant and increased use of tomography, the prevalence of incidental papillary thyroid microcarcinomas (PTCm) is also very high in the country, which might affect the decision to perform surgery [16].

### Malignancy rate evaluation

All FNA cases with an ND result were compared to the corresponding histologic diagnoses. The malignancy rate was estimated by dividing the total number of ND patients by the number of malignant cases. Incidental malignancies that did not correspond to the clinical lesion were not counted as malignant lesions.

### Statistical analysis

We performed univariate regression analysis for well-known risk factors such as age, gender, number of nodules, size of the nodule at the time of ultrasound, and consistency of the lesion upon macroscopic examination. Statistical analyses were performed using NCSS (Number Cruncher Statistical System) 2007 Statistical Software (Utah, USA). To compare qualitative variables, Yates continuity correction and Fisher’s exact tests were used. For comparing tumor size and age, a Mann Whitney U test was applied. An overall 5% type-1 error level was used to infer statistical significance, with the significance level of the P value set at p<0.05.

### Ethics statement

The study approved by the Intuitional Review Board of Haydarpasa Education and Research Hospital for all aspects and all gave informed consent.

## Results

### Study cohort and patient characteristics

**Fig 2:**
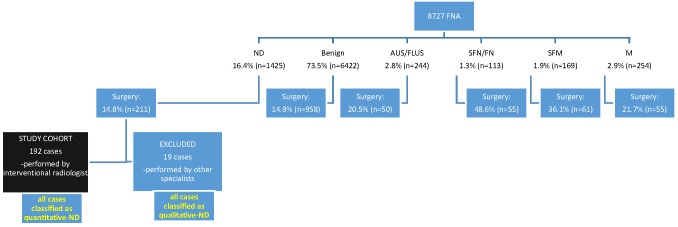
Distribution of FNA cases during the study period.

During the study period, 8,727 thyroid FNAs were identified, of which 1390 were managed with thyroidectomy. The distribution of cytological diagnoses is shown in Fig 2.

Of the 1425 ND samples, 211 had subsequent thyroidectomy. One hundred and ninety-two cases that were performed by the interventional radiology department with US guidance and on-site evaluation comprised the study cohort. The remaining 19 FNAs did not fulfill these criteria, and were thus excluded. From the 192 cases, four patients had a repeat FNA of the same nodule that repeatedly yielded an ND result.

In the cohort of 192 ND cases, 81.3% (n = 156) were women and 18.7% (n = 36) were men with a mean age of 50.6 years (range 20–87 years). The average nodule size was 33 mm (range 3–95 mm). Seventy-six percent (n = 146) were solid, whereas 24% (n = 46) were complex (solid and cystic). None of these nodules were completely cystic upon ultrasound examination. All 192 cases were placed in the quantitative ND group after re-evaluation of the slides, and none were ND for qualitative reasons.

### Cyto-histopathologic correlation and malignancy rate of the ND category

There were nine malignant cases after final histopathologic diagnoses of the 192 ND FNAs, and the malignancy rate was calculated as 4.7%. Five papillary microcarcinomas (PTCm), three papillary thyroid carcinomas (PTC), and one medullary thyroid carcinoma (MTC) with an average size of 14 mm (range 5–40 mm) constituted the malignant histopathologic diagnoses (Table 1).

**Table 1:** Distribution of histopathologic diagnoses.

**Benign 95.3% (n = 183)**	
Follicular nodular disease	*55*.*7% (n = 102)*
Adenomatous nodule	*14*.*8% (n = 27)*
Diffuse hyperplasia	*9*.*9% (n = 18)*
Chronic lymphocytic thyroiditis	*3*.*8% (n = 7)*
Follicular adenoma	*13*.*1% (n = 24)*
Oncocytic adenoma	*1*.*6% (n = 3)*
Encapsulated follicular variant of papillary thyroid carcinoma (noninvasive follicular thyroid neoplasms with papillary-like nuclear features” (NIFTP)	*1*.*1% (n = 2)*
**Malignant 4.7% (n = 9)**	
Papillary thyroid carcinoma	*33*.*3% (n = 3)*
Papillary thyroid microcarcinoma	*55*.*5% (n = 5)*
Medullary carcinoma	*11*.*1% (n = 1)*

The patient characteristics of the nine malignant cases are shown in Table 2. No gender or age differences were observed between benign and malignant nodules. Mean nodule size was greater in the benign group (34 mm vs 14 mm), whereas malignant cases exhibited multiple thyroid nodules more frequently (p>0.05; Table 3). However, none of the nodule (size, consistency and number) or patient factors (gender and age) were found to be predictive for malignancy (Table 4).

**Table 2:** Patient and nodule characteristics of malignant cases.

Case number	Age	Gender	Number of nodules	Cystic/Solid	Nodule size (mm)	Histopathology
1	60	M	Multiple	Solid	8	PTCm
2	44	F	Multiple	Solid	6	PTCm
3	24	F	Multiple	Solid	10	PTCm
4	59	M	Multiple	Solid	8	PTCm
6	82	F	Multiple	Solid	15	MTC
7	30	F	Solitary	Cystic	15	PTC
8	28	F	Solitary	Solid	40	PTC
9	66	M	Multiple	Cystic	27	PTC
11	32	F	Multiple	Solid	5	PTCm

F: Female; M: Male; PTC: Papillary thyroid carcinoma; EFV-PTC: Encapsulated follicular variant papillary thyroid carcinoma; MTC: Medullary thyroid carcinoma; PTCm: Papillary thyroid microcarcinoma

**Table 3:** Comparison of nodule and patient characteristics in benign and malignant cases.

	Benign (n = 183)	Malignant (n = 9)
*Age*, *years*, *mean (range)*	50,74 (20–87)	47,22 (24–82)
*Gender*		
*Female*	81.4% (n = 149)	77.8 (n = 7)
*Male*	18.6% (n = 34)	22.2 (n = 2)
*Consistency*		
*Solid*	76.0% (n = 139)	77.8% (n = 7)
*Cystic*	24.0% (n = 44)	22.2% (n = 2)
*Nodule size*, *mm*, *mean (range)*	34,0 (3–95 mm)	14,0 (5–40 mm)
*Number of nodules*		
*Solitary*	35.0% (n = 64)	22.2% (n = 2)
*Multiple*	65.0 (n = 119)	77.8% (n = 7)

**Table 4:** Risk factors for malignancy.

	Odds ratio	95% CI for odds ratio	P value*
Size (≥4cm / ≤ 4 cm)	*1*,*056*	*1*,*019–1*,*095*	*1*.*000*
Gender (female/male)	*1*,*262*	*0*,*249–6*,*296*	*0*.*677*
Consistency (cystic/solid)	*1*.*108*	*0*,*222–5*,*530*	*1*.*000*
Age (≥51 / < 50 years)	*0*,*864*	*0*,*225–3*,*323*	*1*.*000*
No. of Nodules (Multiple/single)	*1*.*882*	*0*,*380–9*.*329*	*0*.*721*

## Discussion

Since Walfish et al. eloquently stated the usefulness of US-guided percutaneous FNA in the management of thyroid nodules in 1976, it has been proven to be an accurate and cost-effective method [18]. However, an ND result remains a limitation for thyroid FNA, and it was demonstrated as the most frequent source of false negative diagnoses [19–21].

The risk of malignancy for an ND result is difficult to assess because only a small subset of ND nodules will be resected. Hence, the reported malignancy rates demonstrate significant variation, ranging from 2% to 51% in the literature [6, 8–15] (Table 5). In the present study, the malignancy rate of the ND category was evaluated in relation to histopathologic diagnoses and determined to be 4.7%, which is near the low end of other reported series.

**Table 5:** Non-diagnostic and malignancy rates for thyroid FNA in the literature.

Author	Year	Total FNA (n)	Total ND FNAs (n)	ND FNA/Total FNA (%)	Surgical follow-up (n)	Malignancy rate in the ND category (%)
MacDonald and Yazdi et al. [22]	1996	NA	NA	91	NA	2%
Al Maqbali et al. [23]	2014	1657	264	16%	68	(12/68) 18%
Yoon et al. [24]	2010	22754	3701	16.3%	230	(101/230) 43.9%
Schmidt et al.[25]	1997	345	59	17.1%	21	(4/21) 5.1%
Baloch et al. [8]	2003	3007	237	7%	53	(27/53) 51%
Woo et al. [15]	2015	1203	84	6.98%	51	(36/51) 70.6%
Yang et al. [19]	2007	4703	488	10.4%	46	(5/49) 10.8%
Renshaw et al. [12]	2010	7089	1671	23.5%	235	(47/235) 20%
Andre R Le et al. [14]	2015	-	197	25%	49	(6/49) 12.2%
Deandrea et al. [26]	2010	927	-	-	51	(3/51) 5.8%
Piana et al. [27]	2011	18359	2230	12%	96	(23/96) 24%
M.L Richards et al. [28]	2008	241	51	21%	51	(7/51) 14%
Seningen JL et al. [9]	2010	1945	180	9.3%	180	(25/180) 14%
This study	2015	9020	192	6.8&	1390	(9/192) 4.7%

FNA: Fine-needle aspiration; ND: Non-diagnostic

MacDonald and Yazdi et al. [22] reported the lowest malignancy rate to date in a cohort of 91 ND FNAs with histopathologic follow-up, which was 2%. However, the malignancy rate would have been 12% if the 9 PTCm that were not incidental were counted as malignant in this study. Similarly, the study by Al Maqbali et al. also demonstrated the importance of the method used for malignancy rate calculation [23]. Al Maqbali et al. estimated the malignancy rate to be 4.5% by dividing total number of all FNAs (with or without surgical follow-up) by the number of malignant cases [23]. Impressively, when the malignancy rate did not include nodules without surgical follow-up, which was the same method used in the present study, the malignancy rate increased four-fold to 18%.

In another study by Schmidt et al., the malignancy rate was reported as 5% (3/59) with the histopathologic result as the final diagnosis [25]. Baloch et al. reported a 51% malignancy rate for the ND category, which was quite high when compared to the literature [8]. Similar to our approach, only quantitative ND cases were included in the study by Baloch et al., which might be the reason for the highest malignancy rate. All of the aforementioned studies applied BSRTC or Goellner’s adequacy criteria for specimen evaluation, except Al Maqbali et al. employed the Royal College of Pathologist’s criteria [4, 23]

Regardless of the different criteria that were used, in contrast with the present study, none of the aforementioned studies specifically referred to the cause of ND aspirations in their study population. Likewise, clinicians who perform the FNA, ultrasound guidance, and presence of on-site evaluation were not uniform between studies or even within studies. All these factors contribute to the wide range of malignancy rates in the literature, and complicate the comparison of the results because too many parameters, including the FNA procedure and the malignancy rate estimation method, should be taken into consideration.

Interestingly, Moon et al. assessed a malignancy rate of 11.5% in a cohort of 104 ND FNAs based on both surgical results and defined clinical follow-up data (after two consecutive ND results, thyroid nodules with two consecutive benign cytology results, one benign cytology result and no change on follow-up US for more than 12 months, and decrease in size on follow-up US were regarded benign). They estimate the malignancy rate for sonographically suspicious and benign nodules as 25.7% and 4.3%, respectively [20].

The literature has limited data on whether patient demographics, clinical findings, and/or radiologic findings could predict malignancy. Moon et al. determined the malignancy rate to be 13% for solid nodules, whereas it was 8.6% for cystic nodules. Contrary to general beliefs, they claimed that one should be careful to have a tendency to perform surgery based on the solid nature of the nodule due to the fact that solid nodules were mostly benign (87%) [19]. Our data revealed that the malignancy rate was higher in solid nodules when compared to cystic nodules (Odds ratio: 1.960; 95% CI for odds ratio: 0.598–6.421; p *=* 0.515). However, 94.4% of solid nodules were benign in the present study, which supports the suggestion of Moon et al.

Moreover, Moon et al. and Rosario et al. claimed that radiation exposure, family history, number of nodules (e.g., solitary vs. clustered), and size (>3 cm) were not predictive of a malignant result [20, 29]. In contrast, Al Maqbali et al.[23] revealed that a nodule size >4 cm, multiplicity, male gender, and solid nature can predict malignancy, even though the results were not statistically significant. McHenry et al. also observed that male gender was significantly higher in malignant cases [11]. Woo et al. analyzed sonographic findings and reported that only hypoechogenicity was related with malignancy via multivariate analysis [15]. Surprisingly, in the current study, nodule size was found to be larger in the benign group and malignant cases were presented as multiple nodules more often, although these findings were not statistically significant. Additionally, male gender and sonographic features of the nodule (solid or complex) were not related to a malignant diagnosis.

Previous studies have referred to the malignancy ratio of repeated ND results, which also demonstrates a wide range between 0% and 51% [12, 15, 16, 19]. The purpose of the present study was to analyze the malignancy rate of initially ND FNAs by reevaluating the cases and subcategorizing them as “quantitative” or “qualitative” on the basis of BSRTC adequacy criteria; thus, defining the role of repeat FNA in ND aspirations was beyond the scope of this paper. Recently, Woo et al. pointed out that BRAF mutational analysis could be informative for FNA samples that were inadequate according to “qualitative” criteria. The findings of the present study showed that thyroid FNAs that were performed by a core group of specialists under ultrasound guidance with on-site evaluation were all “quantitatively” ND. However, for institutions where the “qualitative” ND samples yield a significant clinical problem, as ATA guidelines were also referred, mutational analysis could aid in patient management [1]. A major limitation of the present study was the exclusion of ND cases that did not have a thyroidectomy, which may reflect a selection bias in malignancy rate estimation. Furthermore, all aspirations were performed by an interventional radiologist with the presence of on-site evaluation. Although the absolute effect of these inclusion criteria cannot be determined, it would be expected that both the ND aspiration ratio and cause of inadequacy would be affected by this standardization.

The present study demonstrated that the ND category is still a significant limitation of the FNA method, with 15.2% of initial FNAs defined as ND and 4.7% of the ND nodules showing malignancy. In addition, none of the patient or nodule characteristics were associated with malignancy in nodules with ND results. In view of previous reports and the present study, we conclude that standardization of the FNA procedure, malignancy rate estimation based on cyto-histopathologic correlation, and identifying the cause of inadequacy could be informative for the future management protocols.

## Supporting Information

S1 File Data Set of the manuscript. (XLSX)
